# Treatment strategies for hormone receptor-positive, human epidermal growth factor receptor 2-positive (HR+/HER2+) metastatic breast cancer: A review

**DOI:** 10.3389/fonc.2022.975463

**Published:** 2022-12-23

**Authors:** Ran Ran, Yingying Ma, Hui Wang, Jin Yang, Jiao Yang

**Affiliations:** Department of Medical Oncology, The First Affiliated Hospital of Xían Jiaotong University, Xían, China

**Keywords:** advanced breast cancer, combination therapy, endocrine therapy, HR+/HER2+, human epidermal growth factor receptor 2-positive, hormone receptor-positive, resistance, clinical trials

## Abstract

Hormone receptor-positive HER2-positive (HR+/HER2+) metastatic breast cancer (MBC) is a unique subtype of breast cancer. Most current guidelines recommend that combination regimens based on anti-HER2 therapy should be used as first-line treatment for HER2+ MBC, irrespective of HR status. Endocrine therapy can be applied as maintenance therapy for patients who are intolerant to chemotherapy or post-chemotherapy. Increasing evidence suggests that complex molecular crosstalk between HR and HER2 pathways may affect the sensitivity to both HER2-targeted and endocrine therapy in patients with HR+/HER2+ breast cancer. Recent research and clinical trials have revealed that a combination of endocrine therapy and anti-HER2 approaches without chemotherapy provides along-term disease control for some patients, but the challenge lies in how to accurately identify the subsets of patients who can benefit from such a de-chemotherapy treatment strategy. In this review, we aim to summarize the results of preclinical and clinical studies in HR+/HER2+ MBC and discuss the possibility of sparing chemotherapy in this subgroup of patients.

## 1 Introduction

Breast cancer is the most common cancer worldwide, with 2.3 million new diagnoses in 2020, surpassing lung cancer for the first time and accounting for approximately 25% of malignancies in women (1). Human epidermal growth factor receptor 2-positive (HER2+) breast cancer accounts for about 20%–25% of all breast cancer cases and nearly 50% of these HER2+ cases also express hormone receptors (HRs), including estrogen receptor (ER) and/or progesterone receptor (PR) (2). Several studies have shown that patients with HR+/HER2+ breast cancer are more likely to exhibit resistance to anti-HER2 and endocrine therapy and have a worse prognosis than patients with hormone receptor-positive HER2-negative (HR+/HER2-) disease. This review will focus on the molecular biological characteristics, current treatment, and preclinical and clinical research progress in HR+/HER2+ MBC, to explore its heterogeneity and provide more evidence for clinical practice, promoting the development of precise treatment of HR+/HER2+ MBC.

## 2 HR+/HER2+ breast cancer as a special phenotype

It is generally considered that breast cancers can be classified into four molecular subtypes (luminal A, luminal B, HER2-enriched, and basal-like) according to the gene expression patterns (3). Clinically, immunohistochemistry analysis is often used as an alternative method to classify breast cancer into four types (luminal A, luminal B, HER2-enriched, and triple-negative) based on the expression status of ER, PR, HER2, and Ki67 (4). Unlike HR-negative/HER2-positive (HR-/HER2+) breast cancer in which 74% is HER2-enriched subtype, the HER2-enriched subtype, and luminal-like subtype each account for about half of the HR+/HER2+ breast cancer (5). According to molecular intrinsic subtypes, around 70% of HR+/HER2+ tumors are luminal A or B, which are estrogen receptor-dependency, with low HER2/EGFR-pathway activation and a high rate of PIK3CA mutations, that are associated with lower response to anti-HER2 treatment but better prognosis. While about 30% of HR+/HER2+ tumors are HER2-enriched, with relation to high HER2/EGFR-pathway activation, high proliferation rate, and an immune-activated stroma with elevated tumor-infiltrating lymphocytes (TILs) levels (6). Another study revealed that compared with ER-/PR-/HER2+ breast cancers, ER+/PR+/HER2+ breast cancers exhibited different driving events, including lower TP53 mutation rate, lower ERBB2 amplification rate, and higher CCND1 amplification rate (7). These biological differences lead to a different prognostic pattern and treatment sensitivity.

In the patients with HER2+ early breast cancer (EBC), HR+ patients perform a better 5-years disease-free survival (DFS) than HR- patients; however, the recurrence risk may persist longer in HR+ patients, finally resulting in a similar long-term outcome compared to the HR- patients (8). In terms of treatment sensitivity, HR+/HER2+ patients are less sensitive to neoadjuvant chemotherapy plus anti-HER2 drugs with lower pathologic complete response (pCR) rates vs HR-/HER2+ patients (9). A clinical study including 719 patients with HR+ breast cancer demonstrated that the clinical benefit rate (CBR) of endocrine therapy was significantly lower in the high serum HER2 group than in the low HER2 group (23% vs 45%, *p* < 0.0001), with a significant reduction in time to disease progression (TTP) and overall survival (OS) (*p* < 0.0001) (10). A meta-analysis of 12 studies (n = 2379) verified that HER2+ MBC is less responsive to endocrine treatment, with an overall relative risk (RR) of 1.42 (*p* < 0.0001) (11). The studies mentioned above illustrate that HER2 overexpression may affect the efficacy of endocrine therapy for HR+ breast cancer. A retrospective study of patients with ER+/PR+/HER2+ breast cancer showed that trastuzumab plus chemotherapy significantly prolonged recurrence-free survival (RFS) and breast cancer-specific survival (BCSS) compared with chemotherapy alone, but no significant survival benefit was obtained in the subgroup with > 50% expression of both ER and PR (12). Results from the HERA study and NSABP B-31 study also showed a reduced survival benefit from the addition of trastuzumab treatment in patients with high ER expression (13, 14). The above studies illustrate that ER also impacts the efficacy of anti-HER2 targeted therapy. A study reported the 10-year annual hazard of recurrence in 1260 patients with HER2+ EBC who did not receive anti-HER2 adjuvant therapy, a lower early risk of relapse was observed in HR+/HER2+ patients compared to HR-/HER2+ patients, and HR+ patients had a stable and sustained annual risk of relapse (15). In summary, there exists remarkable heterogeneity in HR+/HER2+ breast cancer that affects the treatment response and prognosis of patients. Identifying patients with different subtypes and giving individualized treatment is essential to improve the survival of patients with HR+/HER2+ breast cancer.

## 3 HR/HER2 crosstalk and treatment resistance

**Figure 1:**
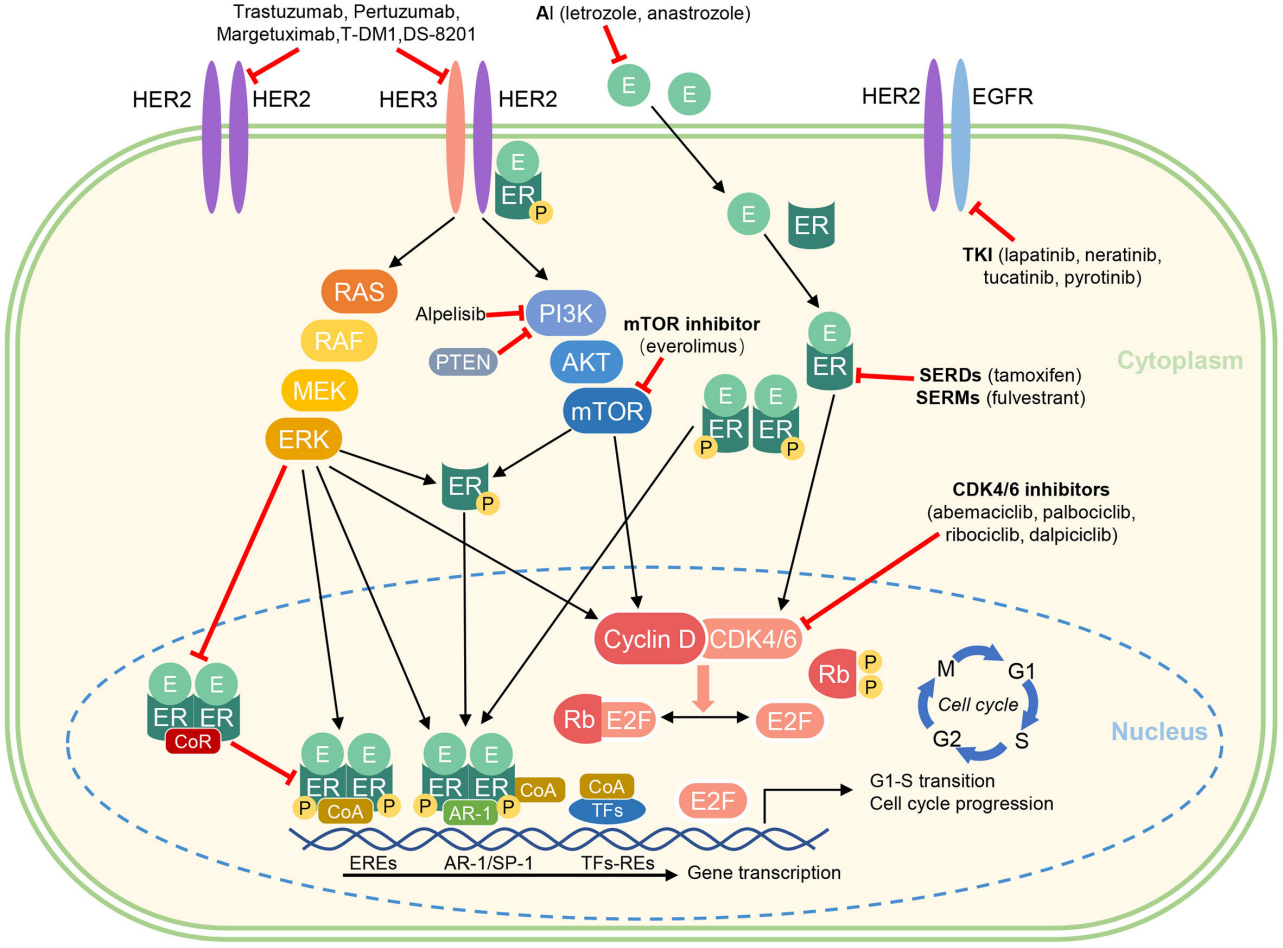
Crosstalk between ER and HER2 signaling and pharmacological mechanism of endocrine therapies and targeted agents.

In clinical practice, HR+/HER2+ breast cancers tend to become resistant to endocrine therapy and/or HER2-targeted therapy over time. A large number of studies have shown that the complex molecular signaling crosstalk between the ER/PR and HER2 signaling pathways may contribute to treatment resistance and will promote tumor progression (16, 17). A study of HER2+ cell lines showed increased expression levels of ER or its downstream signaling targets following treatment with lapatinib and trastuzumab, indicating ER signaling as a survival mechanism reducing sensitivity to HER2-blockade (18, 19). Giuliano et al. found that 18% of HER2+ tumors that were initially ER- were converted to ER+ after2 weeks of neoadjuvant therapy using lapatinib, further demonstrating the interconnection of ER and HER2 pathways (20). Anti-HER2 therapy may reactivate estrogen receptor signaling as a mechanism of resistance. In contrast, theoveractivity of HER2 including mitogen-activated protein kinase (MAPK) and phosphatidylinositol 3inase (PI3K/AKT) may interfere with ER expression and activity, thus representing a potential escape pathway for HR+ breast cancer overexpression of HER2and leading to a new state of endocrine resistance (21). Multiple clinical trials demonstrated that HR+/HER2+ breast cancer patients have a reduced sensitivity to neoadjuvant chemotherapy plus anti-HER2 agents and a lower chance to achieve a pCR compared with HER2+/HR- patients (9). The combination of ER/HER2-targeted therapies may improve prognosis. Therefore, regimen targeting pathways implicated in this crosstalk appear to be a possible strategy to delay or reverse drug resistance (22). Receptor mechanisms involved in the progression of breast cancer are summarized in **Figure 1**.

AI, aromatase inhibitor; AKT, protein kinase B; CDK, cyclin dependent kinase; CoA, coactivator complex; CoR, corepressor complex; E, estradiol; EGFR, epidermal growth factor receptor; ER, estrogen receptor; EREs, estrogen receptor elements; ERK, extracellular signal-regulated kinase; HER2, human epidermal growth factor receptor 2; P, phosphorylation; PI3K, phosphatidylinositol 3-kinase; mTOR, mammalian target of rapamycin; Rb, Retinoblastoma protein (tumor suppressor protein); RE, response elements; SERD, selective estrogen receptor degrader; SERM, selective estrogen receptor modulator; TKI, Tyrosine kinase inhibitors; TFs, transcription factors [e.g. activator protein 1 (AP-1), specificity protein 1 (SP-1), and E2 factor (E2F)].

## 4 Treatment of HR+/HER2+ metastatic breast cancer

HER2-targeted therapy has considerably improved the survival outcomes of patients with HER2+ breast cancer. In the era of tailored treatments, the most appropriate therapy for HR+/HER2+ MBC is a matter of debate. Major guidelines recommend HER2-targeted therapy as the basis of treatment for HR+/HER2+ breast cancer in combination with chemotherapy or endocrine therapy, and the combination of drug regimens and how to select the appropriate population are the main concerns of clinical research (23). A number of previous clinical trials, summarized in **Table 1**, have been conducive in identifying new combination treatments for patients with HR+/HER2+ MBC.

**Table 1:** Previous clinical trials treating HR+/HER2+ MBC.

Clinical trial	Phase	Cohort size	Treatment Regimen	Outcome (months)
**ET ± single HER2-targeted therapy**				
TAnDEM (24)	III	207	Trastuzumab + anastrozole vs anastrozole	PFS: 4.8 vs 2.4, *HR* = 0.63, *p* =0.0016
eLEcTRA (25)	III	57	Trastuzumab + letrozole vs letrozole	PFS: 14.1 vs 3.3, *HR* = 0.67, *p* = 0.23
EGF30008 (26)	III	219	Lapatinib + letrozole vs letrozole	PFS: 8.2 vs 3.0, *HR* =0.71, *p* =0.23
**ET + dual HER2-targeted therapy**				
PERTAIN (27)	II	258	Pertuzumab + trastuzumab + AI vs trastuzumab + AI	PFS: 18.9 vs 15.8, *HR* =0.65, *p* =0.007
ALTERNATIVE (28)	III	355	Lapatinib + trastuzumab + AI vs lapatinib + AI vs trastuzumab + AI	PFS: 11.0 vs 8.3 vs 5.7
**HER2-targeted therapy + ET or CT**				
Sysucc-002 (29)	III	392	Trastuzumab + ET vs trastuzumab + CT	PFS: 19.2 vs 14.8
**Dual HER2-targeted therapy + ET**± **CT**				
PERNETTA (30)	II	210	Trastuzumab + pertuzumab + ET vs trastuzumab + pertuzumab + ET + CT	2-year OS: 75.0% vs 74.2%
**CDK4/6 inhibitor**				
monarcHER (31)	II	237	Abemaciclib+ fulvestrant + trastuzumab vs abemaciclib + trastuzumab vs standard-of-care chemotherapy + trastuzumab	PFS: 8.3 vs 5.7 vs 5.7
PATRICIA (32)	II	30	Palbociclib + trastuzumab + letrozole vs palbociclib + trastuzumab	6-month PFS: 42.8% vs 46.4%
**ADC**				
DESTINY-Breast03 (33)	III	272	Trastuzumab Deruxtecan vs Trastuzumab Emtansine	PFS: 22.4 vs 6.9 months, *HR* =0.32, 95%, *p* <0.001

AI, aromatase inhibitor, CDK, cyclin-dependent kinase, CT, chemotherapy, ET, endocrine therapy, HER2, human epidermal growth factor receptor 2, HR, hormone receptor; *HR*, hazard radio;l MBC, metastatic breast cancer; OS, overall survival; PFS, progression-free survival.

### 4.1 Endocrine therapy combined with single anti-HER2 therapy

To overcome endocrine or anti-HER2 therapy resistance in HR+/HER2+ MBC patients, several studies have evaluated the therapeutic effect of the combination of endocrine therapy and a single anti-HER2 drug. In the TAnDEM trial (24), researchers evaluated the efficacy of trastuzumab combined with anastrozole as a first-line treatment for HR+/HER2+ MBC. The trastuzumab plus anastrozole arm (n = 103) experienced a significantly improved progression-free survival (PFS), with a median PFSof 4.8 months versus 2.4 months in the anastrozole alone arm (n = 104). In addition, in patients with HR+/HER2+ MBC confirmed by central evaluation (n=150), median PFS was 5.6 and 3.8 months in the trastuzumab plus anastrozole and anastrozole alone arms,respectively (*p* = 0.006). The eLEcTRA study (25) was a similar prospective, multicenter, randomized controlled study, comparing the first-line efficacy of letrozole plus trastuzumab and letrozole monotherapy in patients with MBC. The results also showed that letrozole combined with trastuzumab significantly prolonged PFS compared with letrozole alone. These findings indirectly suggested that endocrine therapy alone is more likely to induce drug resistance in HR+/HER2+ breast cancer patients.

Another therapeutic method to inhibit HER2-mediated signal transduction is to use small-molecule tyrosine kinase inhibitors (TKI) targeting HER2 and EGFR intracellular kinase domains, such as lapatinib, neratinib, tucatinib,and pyrotinib. In the phase III EGF30008 trial (26), researchers randomized patients with HR+ MBC into two groups (letrozole plus lapatinib or placebo). In the predetermined HR+/HER2+ subgroup (n = 219), the median PFS in the letrozole plus lapatinib and letrozole alone groups were 8.2 and 3.0 months (*HR* = 0.71; *p* = 0.019), with a significant reduction in the risk of progression for the combination. However, overall survival (OS) was not significantly improved (33.3 vs 32.3 months; *HR* = 0.74; *p* = 0.113).

The results of the above-mentioned studies showed that the combination of hormonal and single HER2 (trastuzumab or TKI) blockade led to a mild improvement in PFS but did not turn into significant OS benefit, which suggests that the interruption of the crosstalk between HER2 and ER might be effective only in a few cases or for a short duration. This strategy could be relegated to patients who are intolerant to chemotherapy or as an empirical maintenance strategy after chemotherapy.

For HR+/HER2+MBC patients, there is little research evidence to support which partner strategy is the more preferred, endocrine therapy or chemotherapy, in combination with anti-HER2 therapy as the first-line management. A real-world study (34) based on the US National Cancer Database showed that among the enrolled 6234 patients from 2010 to 2015, 60% of them received endocrine therapy while 40% underwent chemotherapy as a combinative therapy with anti-HER2 therapy in first-line treatment. The results showed that endocrine therapy significantly improved the median OS rate (56.0% vs 46.8%, *p* = 0.004) and the 5-year survival rate (47.5% vs 39.8%, *p* < 0.001) compared with chemotherapy when added to anti-HER2 therapy. This is thefirst real-world study to compare chemotherapy and endocrine therapy in patients with HR+/HER2+ MBC. However, this study is observational and there may be a mismatch between the two groups, such as a lower tumor load, slower disease progression, and a larger post-menopausal population in those who opted for endocrine therapy. Therefore, a further statistical inference may require a prospective randomized controlled clinical study.

The SYSUCC-002 study (29) is the first randomized phase III study to compare the efficacy and safety of trastuzumab plus endocrine therapy or chemotherapy as the first-line treatment for HR+/HER2+ MBC in a head-to-head manner. The median PFS of the two groups was 19.2 months and 14.8 months (*p* < 0.0001), respectively, which confirmed that trastuzumab plus endocrine therapy was non-inferior to trastuzumab plus chemotherapy but with reduced toxicity. Subgroup analyses showed that patients with disease-free intervals (DFI) longer than 24 months benefited more from endocrine therapy than patients with DFI less than 24 months. However, the majority of the eligible population (80.9%) were both ER and PR positive. 59.4% had visceral involvement and 47.9% had recurrent disease over 24 months since the diagnosis of primary breast cancer. The enrollment of a low-risk population was a possible determinant of the study meeting the primary study endpoint. Hence, HER2-targeted therapy combined with endocrine therapy may be a more appropriate first-line treatment option for low-risk patients with HR+/HER2+ MBC, illustrating a potential modality shift toward a chemotherapy-sparing regimen.

### 4.2 Endocrine therapy combined with dual anti-HER2 therapy

CLEOPATRA study (35) demonstrated that trastuzumab and pertuzumab plus a taxane could further improve PFS and OS in patients with HER2+ MBC, regardless of HR status, which makes dual HER2-targeted therapy regimen the preferred standard of the first-line treatment of HER2+ MBC. In the second- and subsequent-line management of HR+/HER2+ MBC, HER2CLIMB trial revealed that the addition of tucatinib to trastuzumab and capecitabine demonstrated a significant improvement in OS, with a median survival benefit of 5.5 months (OS: 24.7 vs. 19.2 months; *HR* = 0.73; *p* = 0.004). There was consistent benefit across subgroups, including patients with HR+ disease and, importantly, in patients with brain metastases (36)

In the PERTAIN study (27), 258 patients with HR+/HER2+ MBC were randomly assigned to aromatase inhibitor (AI) and trastuzumab plus pertuzumab or placebo. The result showed an improved PFS with the three-drug combination (18.89 vs 15.80 months, *HR* =0.65, *p* = 0.007). In the two groups, the PFS of patients who did not receive induction chemotherapy was 21.72 and 12.45 months (*p* = 0.011), while the PFS of patients who received induction chemotherapy was 16.89 and 16.85 months (*p* = 0.163). Therefore, endocrine therapy combined with trastuzumab and pertuzumab could further improve the PFS of patients with good tolerance. In the ALTERNATIVE study (28), 355 patients with HR+/HER2+ MBC who had received prior trastuzumab,endocrine therapy, and chemotherapy were enrolled. The results suggested an improvement in PFS by the addition of lapatinib to AI and trastuzumab. The median PFS in the lapatinib plus trastuzumab and AI arm was 11.0 months, which is comparable to the results of dual anti-HER2 inhibition combined with chemotherapy, as observed in the EMILIA or PHEREXA trials.

RegistHER (37) study is a prospective observational cohort to examine the first-line treatment patterns and outcomes in 530 patients with HR+/HER2+ MBC in a real-world setting. The results showed that the PFS of patients receiving sequential endocrine therapy after trastuzumab plus chemotherapy was longer compared with those receiving trastuzumab plus chemotherapy or trastuzumab plus endocrine or single endocrine therapy (21.3 vs 9.5 vs 13.8 vs 4.8 months). Taken together with the PERTAIN study, it can be inferred that the survival benefits of combined chemotherapy with single target plus endocrine therapy are superior to those of single HER2 blockade plus chemotherapy or endocrine therapy. However, there is no survival benefit or even less with combined chemotherapy when receiving a double-blocking escalated anti-HER2 therapy. The phase II PERNETTA trial (30) compared the survival of patients with HR+/HER2+MBC who received first-line treatment with pertuzumab and trastuzumab with or without chemotherapy (two groups were treated with second-line TDM1 therapy after relapse). The 2-year median OS was similar in both groups, 75.0 and 74.2 months, respectively. This reflects that dual HER2-targeted therapy plus chemotherapy did not improve OS in the general population. Besides, the side effects of the chemotherapy-free group were less. Therefore, enhanced anti-HER2 therapy can further improve PFS and is well tolerated. Endocrine therapy combined with dual anti-HER2 therapy may allow patients to avoid chemotherapy and could become the first-line choice for the part of these patients.

### 4.3 Anti-HER2 therapy combined with additional therapy

Several trials have examined anti-HER2-based therapy in combination with different targeted agents in patients with HER2+ MBC, with variable results in the HR+/HER2+ subgroup.

#### 4.3.1 Cyclin-dependent kinase 4/6 inhibitor

CDK4 and CDK6 are critical regulators of cell-cycle progression. CDK4/6 inhibitors can block the transition of tumor cells from G1 to S phase by selectively inhibiting the function of CDK4/6 (38). CDK4/6 inhibitors have now become a promising strategy for HR+/HER2+ breast cancer treatment as it is the downstream of the ER and HER2 pathways, as well as many other cellular pathways inducing resistance to HER2-targeted therapies (39). Preclinical studies have reported that CDK 4/6 inhibitors can regain the sensitivity to anti-HER2 therapies. The latest ER+/HER2+ cell line experiment showed that the combination of fulvestrant plus dual HER2-blockade (trastuzumab and pertuzumab) plus palbociclib (PFHPert) could be bypassed by co-targeting Rb to eliminate the escape mechanism induced by ER/HER2 crosstalk, promoting the transition from cell quiescence to sustained senescence (40). Currently, there are four CDK4/6 inhibitors on the market, including palbociclib, ribociclib, abemaciclib, and dalpiciclib. A combination of CDK4/6 inhibitors, hormonal therapy, and HER2-targeted agents has demonstrated promising efficacy in several phase I/II trials. The phase II monarcHER trial (31) showed that in patients with HR+/HER2+ MBC, the combination of abemaciclib, fulvestrant, and trastuzumab significantly improved PFS versus standard-of-care chemotherapy plus trastuzumab (8.3 vs 5.7 months, *HR* = 0.67, *p* = 0.051) in the third or later line settings. Moreover, a similar study (NCT03054363) of tucatinib, Palbociclib, and letrozole showed considerable anti-tumor activity and tolerable safety in patients with two lines of prior therapy for HR+/HER2+ MBC, even in those with brain metastases (38%). In the phase II PATRICIA study (32), the efficacy of palbociclib plus trastuzumab was assessed in patients with HER2+ MBC who had received prior 2-4 lines of anti-HER2 treatment. Cohort B included patients with HR+cancer who were randomly divided into two groups to receive palbociclib plus trastuzumab plus letrozole or palbociclib plus trastuzumab. The PFS rates at 6 months (PFS6) in the two groups were 46.4% and 42.8%, respectively. In addition, 59 tumors (83.1%) were tested for Prediction Analysis of Microarray (PAM50) and the luminal subtype was independently associated with a longer PFS compared with the non-luminal subtype. These results have provided an option for patients with multi-line therapy to avoidchemotherapy. Dalpiciclib is the first original Chinese CDK 4/6 inhibitor approved for HR+/HER2- MBC. Preclinical studies have shown that dalpiciclib can overcome resistance to HER2-targeted and endocrine therapy in ER+/HER2+ breast cancer cell lines (41), and synergistic antitumor effects combined with pyrotinib (42). Pyrotinib is an irreversible pan-ERBB inhibitor, has shown promising antitumour activity, and acceptable tolerability (43). In 2021, the ASCO annual meeting published the phase Ib LORDSHIPS trial (44) of dalpiciciclib and pyrotinib plus letrozole in the first- or second-line treatment for HR+/HER2+ MBC patients. The confirmed objective response rate (ORR) was 66.7% and PFS was 11.3 months. The novel fully oral triplet combination had been proved to be safe and effective, potentially providing a total oral chemotherapy-free regime for patients with HR+/HER2+ MBC. The planned dose-expansion phase II study is ongoing. The above studies suggest that a triple combination of a CDK4/6 inhibitor, endocrine therapy, and HER2-targeted may be an alternative active and effective treatment option in heavily pretreated patients with HR+/HER2+ MBC.

#### 4.3.2 Fulvestrant

Fulvestrant has been chosen as the preferred endocrine partner in combination with anti-HER2 agents and CDK4/6 inhibitors either in the early and advanced setting (31, 45). In the phase II NA-PHER2 study (45), the combination of pertuzumab, trastuzumab, fulvestrant, and palbociclib achieved an objective response and a pCR rate in 97% and 27% of patients with HR+/HER2+ BC, respectively. The HERMIONE 9 (46) is an Italian retrospective multicentric study designed to describe the outcomes of HR+/HER2+ MBC patients treated with the combination of fulvestrant and trastuzumab. Among the 86 evaluable patients, researchers observed an mPFS of 12.9 months and a CBR of 78%which favorably compare with those previously reported by the combination of an AI and trastuzumab even when administered in earlier lines. Remarkably, 77% of patients had been treated with ≥ 3 previous therapies for metastatic disease and more than 50% of patients had visceral disease. The results suggested that the combination of fulvestrant and trastuzumab was active in this cohort with a poor prognosis.

#### 4.3.3 Antibody-drug conjugate

Trastuzumab emtansine (T-DM1) is an antibody-drug conjugate (ADC) combining trastuzumab and the cytotoxic microtubule-inhibitor DM1. The EMILIA study (47) demonstrated that T-DM1 significantly prolonged PFS and OS (PFS: 9.6 vs 6.4 months, HR=0.65; *p* < 0.001; OS: 30.9 vs 25.1 months, *HR* =0.68, *p* < 0.001) in patients with HER2+ MBC treated in the second line compared with lapatinib plus capecitabine. Hence, TDM-1 was established as second-line therapy in HER2+ MBC, notably, 55% of these patients were HR+. Trastuzumab deruxtecan (T-DXd, or DS-8201) is an antibody-drug combination comprising trastuzumab and a cytotoxic topoisomerase I inhibitor and has broader antitumor activity than T-DM1 (48). T-DXd was approved for patients with HER2+ MBC based on the results from DESTINY-Breast01. Phase III DESTINY-Breast03 (49) evaluated the efficacy and safety of T-DXd versus T-DM1 in 524 patients with HER2+ MBC that had progressed during or after treatment with trastuzumab and taxane. It is noteworthy that 50% of the enrolled population was HR+. The median PFS was not reached (95% CI: 18.5 to could not be estimated) in the T-DXd group and was 6.8 months (95% CI: 5.6 to 8.2) in the T-DM1 group. The HR+ subgroup analysis showed that PFS significantly improved in the T-DXd group over the T-DM1 group (22.4 vs 6.9 months, *HR* = 0.32, 95% CI: 0.22 to 0.46,*p* < 0.001). The efficacy of DS-8201 is consistent in both HR+ and HR- subgroups, whichis related to the mechanism of ADCs that do not rely on the HER2 signaling pathway. T-DXd has a stronger efficacy than T-DM1 due to its high drug-antibody ratio and bystander effects. Adverse event rates in the two groups were similar. T-DXd demonstrated a highly statistically significant and clinically meaningful improvement in PFS and tolerable with manageable toxicity in heavily pretreated HER2+ MBC patients. DESTINY-Breast03 will establish a breakthrough position of T-DXd in the second-line treatmentof patients with HER2+ MBC. At present, most studies focus on dual anti-HER2 combined with endocrine therapy, including CDK4/6 inhibitors. However, there may be evidence of the superiority of ADCs combined with endocrine therapy in the future, which is worthy of further exploration by researchers.

#### 4.3.4 PI3K/Akt/mTOR pathway inhibitor

The PI3K/Akt/mTOR pathway is located downstream of the site where HER2-targeted drugs act and resistance to HER2-targeted therapy may arise through activation of this pathway. The BOLERO-1 trial (33) assessed the addition of the mTOR inhibitor everolimus to trastuzumab plus paclitaxel as a first-line treatment for patients with HER2+ MBC. The BOLERO-3 trial (50) evaluated the addition of everolimus to trastuzumab plus vinorelbine for trastuzumab-resistant patients with HER2+ MBC. However, both trials showed only a small benefit at the cost of increased toxicity. Interestingly, everolimus seemed to improve PFS more in the HR- subgroup, while no PFS difference was observed in the HR+ subgroup. A biomarker analysis revealed that patients with HER2+ MBC and PIK3CA mutations, PTEN loss, or a hyperactive PI3K pathway achieved the better PFS benefit from everolimus. By contrast, alpelisib (an orally bioavailable, small-molecule, *α* -specific PI3K inhibitor) combined with T-DM1 in heavily pretreated patients with HER2+ MBC (47% of which was HR+) yielded an encouraging ORR as high as 43%, including an ORR of 30% in the T-DM1-resistant patients, and a median PFS of 8.1 months (51). Buparlisib (a pan-class-I PI3K inhibitor) in combination with lapatinib showed preliminary evidence of antitumor activity with a manageable safety profile in heavily pretreated patients with trastuzumab-resistant HER2+ MBC, 50% of whom were HR+ (52). These data indicated that PIK3CA inhibitors play an important role in reversing the resistance to anti-HER2 therapy and provide the theoretical for further studies of PI3K inhibition in refractory HER2+ MBC.

## 5 Exploration of de-escalated strategy and biomarkers

Recently, the biggest challenge is how to select patients for de-escalation approaches, maintaining or increasing pCR rates and survival, with minimal toxicity. Thus, the quest for finding prognostic and predictive biomarkers has been intense.

### 5.1 Pathological complete response rate and PAM50

In HER2+breast cancers, previous neoadjuvant trials of chemotherapy plus anti-HER2 therapy consistently showed lower pCR rates in HR+ versus HR- tumors. Several studies have found the neoadjuvant approach to be an effective step on the path toward de-escalation treatment. In the PerELISA study (53), the expression of Ki67 after 2 weeks of letrozole treatment was used to identify whether the patient was a molecular responder who would benefit from a chemotherapy-free regimen with dual anti-HER2 blockade (trastuzumab and pertuzumab). Patients classified as molecular responders (Ki67 relative reduction > 20% from baseline) continued letrozole and started trastuzumab-pertuzumab for five cycles. Patients classified as molecular non-responders started weekly paclitaxel for 13 weeks combined with trastuzumab-pertuzumab. pCR rate was 20.5% and 81.3% for molecular responders and non-responders, respectively. The authors concluded that Ki67 reduction after short letrozole exposure can identify patients who can achieve a meaningful pCR rate without chemotherapy. Additionally, PAM50 intrinsic subtype was significantly associated with Ki67 response and pCR. Among molecular responders, the pCR rate was significantly higher in HER2-enriched than in other subtypes (45.5% vs 13.8%, *p* = 0.042). In another study, PAM50 was used to classify HR+/HER2+ breast cancer. Researchers developed a method based on the immunohistochemical detection of STC2, BCL2, and CDCA8 to screen a luminal A subgroup of TPBCs with a better prognosis and less benefit from trastuzumab (7). PAM50 intrinsic subtype further improves our ability to identify a subset of patients who can avoid chemotherapy. Veeraraghavan et al. (54) found a clinical subtype of breast cancer with high HER2 amplification and intact PI3K pathway that is particularly sensitive to HER2-targeted therapies without chemotherapy. Baseline tumors from patients with ER+/HER2+ EBC treated with neoadjuvant endocrine therapy plus lapatinib and trastuzumab for 12 weeks, were tested for HER2 amplification using fluorescence *in situ* hybridization (FISH), HER2 copy number (CN), and FISH ratio and PI3KCA pathways status defined by PI3KCA mutation or PTEN levels. Thirteen of the 56 patients (23%) achieved pCR. None of the 11 patients achieved pCR in case of a low HER2 ratio and/or CN, whereas 13/45 patients (29%) with higher HER2 ratio and/or CN attained pCR. Higher pCR rates were observed in patients with tumors expressing high PTEN or wild-type PIK3CA (intact PI3K pathway) and in patients with high HER2 ratio and intact PI3KCA pathway (39% and 44%).

The above results indicate that the combination of intrinsic subtype and pCR may further improve more personalized treatment.

### 5.2 The strength of HR expression

A multicenter retrospective analysis (12) found that the different levels of HR expression affected the effect of endocrine therapy. When the expression rate of ER or PR was less than 50%, the biological behavior of HR+/HER2+ breast cancer is more similar to HER2-overexpressing breast cancer. These patients will likely benefit more from chemotherapy plus anti-HER2 therapy. On the contrary, when the ER expression rate is more than 50%, these patients benefit more from endocrine therapy. Another retrospective analysis (55) showed that among patients with HR+/HER2+ EBC who received trastuzumab and chemotherapy, the 5-year and 7-year DFS rates of patients with high HR expression (both ER and PR ≥ 50%) were lower thanthose with low HR expression (ER and/or PR < 50%) (5-year DFS: 75.4% vs 80.8%; 7-year DFS: 67.1% vs 78.0%, *p* < 0.001). Stronger ER/PR co-expression may weaken the beneficial effect of anti-HER2 therapy. These findings may have potential implications for adjusting anti-HER2 treatment based on HR expression intensity.

### 5.3 Imaging examination, exploratory markers, and scoring

Neo-ALL-IN study (56) showed that when patients with ER+/HER2+ EBC received neoadjuvant therapy with a chemo-free regimen (letrozole + lapatinib), high baseline TILs of over 20%, a decrease in the ER Allred score after neoadjuvant treatment, and SUVmax (maximum standardized uptake value, a measure of activity in PET imaging linked to cell viability and proliferation) in baseline FES-PET are to be considered potential biomarkers in these patients. In the PHERGAIN study (57), ^18^F-fluorodeoxyglucose (^18^F-FDG)-PET (^18^F-FDG-PET) was used to evaluate early metabolic responses to neoadjuvant trastuzumab and pertuzumab and the possibility of chemotherapy de-escalation. Patients were randomized into the TCbHP group [docetaxel (T), carboplatin (Cb), trastuzumab (H), and pertuzumab (P)] (A) and dual HER2 blockade group (trastuzumab, pertuzumab plus endocrine therapy or not) (B). Patients assigned to group A completed six cycles of treatment regardless of ^18^F-FDG-PET results. All patients assigned to group B initially received two cycles of trastuzumab and pertuzumab. ^18^F-FDG-PET responders in group B continued this treatment for six further cycles; ^18^F-FDG-PET non-responders in this group were switched to the TCbHP regimen. ^18^F-FDG-PET responders were defined as patients with a reduction of at least 40% from baseline in the SUVmax for all target lesions after 2 cycles of treatment. 227 (79.6%) of 285 patients in group B were ^18^ F-FDGPET responders, of whom 86 (37.9%) of 227 obtained a pCR, and were thus spared postoperative adjuvant chemotherapy. ^18^F-FDG-PET identified patients with HER2+ EBC who were likely to benefit from chemotherapy-free double-target therapy. This strategy might be a valid approach to helping select patients who do not need chemotherapy. IL6ST is a biomarker for ET response (58). In a study presented at the 2020 San Antonio Breast Cancer Symposium (SABCS), higher levels of IL6ST were correlated with active ER signaling and predicted clinical response to neoadjuvant letrozole in ER+/HER2+ EBCs. Lower levels of IL6ST were associated with a lack of response to endocrine therapy and more active HER2 signaling. IL6ST has a potential role in the selection of ER+/HER2+ patients who benefit from HER2-targeted therapy or endocrine therapy alone (59). Kar et al. (60) found that high expression of ESE-1 protein in the nucleus is significantly associated with poor DFS and disease-specific overall survival (DSS) in HR+/HER2+ patients and is an independent prognostic marker for this subgroup. A comprehensive analysis (61) reported the association of the PAM50-based Chemo-Endocrine Score (CES) with pCR and DFS following anti-HER2-based therapy either combined with endocrine therapy or chemotherapy in HR+/HER2+ EBC across seven studies (457 patients). High CES scores may identify patients with a low risk of recurrence despite not achieving a pCR after neoadjuvant therapy, and who may not need treatment escalation such as T-DM1.

Ju et al. (62) constructed a novel marker named rH/E to reflect the relative expression of ERBB2 to ESR1 in each patient. rH/E distinguishes the HER2-enriched subtype breast cancer better than ERBB2 or ESR1 expression alone, which can help to more accurately identify the subgroups of tumor which are predominantly driven by HER2 or ER in the HR+/HER2+ population. For the HER2-driven group, a longer duration and stronger intensity of anti-HER2 therapy may be required. While for the estrogen receptor-driven group, we may be concerned about more effective blockade of ER signaling.

However, further clinical trials are necessary to verify the results of translational studies. Validated prognostic and predictive biomarkers are needed to identify patients who require endocrine therapy alone or in combination with HER2-targeted therapy to avoid over-chemotherapy. The accuracy of screening for the population that would benefit from endocrine therapy by analyzing genomic heterogeneity was higher than that of screening for luminal A based on the ER expression rate. It is anticipated that the PAM50 and other tools have the potential to screen for patients who would benefit from endocrine-based therapies in the future. Further work is needed both in the laboratory and clinical trials to better characterize the potential predictive signaturesin the HR+/HER2+ subgroup. Optimal tailored treatment using biomarkers is underway to be revealed by prospective trials that aim to promote precision medicine in the HR+/HER2+ subgroup.

## 6 Summary

HR+/HER2+ breast cancer is a clinically and biologically heterogeneous disease that differs from other subtypes. Several clinical studies of dual HR and HER2 pathway blockade have generally provided a benefit in PFS for patients with HR+/HER2+ MBC and there was no obvious increase in adverse events in terms of safety. However, it is worth noting that most of the studies have not shown significant improvement in OS and the cost was high. Clinicians should discuss with the patient to weigh the pros and consof each treatment option. Additionally, with the advance of a de-escalating strategy in HR+/HER2+ MBC management, CDK4/6 inhibitors and endocrine therapy plus dual anti-HER2 therapy or novel anti-HER2 drugs might offer an alternative effective and safe chemotherapy-sparing treatment regimen, especially for patients with non-visceral, sensitive to endocrine therapy or older age. In the future, de-chemotherapy, intensive targeted therapy, and endocrine therapy are promising research directions for HR+/HER2+ MBC, meanwhile, the integration of molecular stratification tools and the neoadjuvant setting provides a more individualized treatment platform. There will be an increasing population suitable for endocrine therapy in combination with targeted therapy. Multiple clinical studies are urgently needed to identify biomarkers for the dominant population of de-escalation regimens and guide tailored treatment.

## Author contributions

RR contributed to the study design and performed the work. RR, YM, and HW contributed to data collection. RR, YM performed statistical analysis and interpretation. RR, JiaoY and JinY drafted the manuscript. All authors contributed to the article and approved the submitted version.
